# *Arbutus unedo* L.: Chemical and Biological Properties

**DOI:** 10.3390/molecules191015799

**Published:** 2014-09-30

**Authors:** Maria G. Miguel, Maria L. Faleiro, Adriana C. Guerreiro, Maria D. Antunes

**Affiliations:** 1 IBB-Centro de Biotecnologia Vegetal, Faculdade de Ciências e Tecnologia, Universidade do Algarve Edif. 8, Campus de Gambelas, Faro 8005-139, Portugal; 2 IBB-Centro de Biomedicina Molecular e Estrutural, Faculdade de Ciências e Tecnologia, Universidade do Algarve, Edif. 8, Campus de Gambelas, Faro 8005-139, Portugal

**Keywords:** strawberry tree, plant parts, chemical composition, biological properties, honey, brandy

## Abstract

*Arbutus unedo* L. (strawberry tree) has a circum-Mediterranean distribution, being found in western, central and southern Europe, north-eastern Africa (excluding Egypt and Libya) and the Canary Islands and western Asia. Fruits of the strawberry tree are generally used for preparing alcoholic drinks (wines, liqueurs and brandies), jams, jellies and marmalades, and less frequently eaten as fresh fruit, despite their pleasing appearance. An overview of the chemical composition of different parts of the plant, strawberry tree honey and strawberry tree brandy will be presented. The biological properties of the different parts of *A. unedo* and strawberry tree honey will be also overviewed.

## 1. Introduction

*Arbutus* L. is a genus that belongs to the *Vaccionioideae* subfamily (or *Arbutoideae*, depending on the author), and *Ericaceae* family. In the Mediterranean region there are four species and two hybrids in the genus *Arbutus* L.: *Arbutus unedo* L., *A. andrachne* L (eastern Mediterranean region), *A. pavarii* Pampanini (coasts of Libya), *A. canariensis* Veill. (Canary Islands), *A*. x *andrachnoides* Link (*A. unedo* x *A. andrachne*, in the eastern Mediterranean region), and *A.* x *androsterilis* Salas, Acebes & Arco (*A. unedo* x *A. canariensis*, in the Canary Islands) [1]. *Arbutus unedo* L. is an evergreen shrub that has a circum-Mediterranean distribution, being found in western, central and southern Europe, north-eastern Africa (excluding Egypt and Libya) and the Canary Islands and western Asia, where frost is not very usual and summer dryness is not very intense. In Europe, this species grows in Portugal, Spain, France, Italy, Albania, Greece, Bosnia and Herzegovinia, Croatia, Macedonia, Montenegro, Serbia and Slovenia and in the Mediterranean islands (Balearic, Corsica, Sardinia, Sicily and Crete). It is also able to adapt to conditions of the south-western coast of Ireland [1,2,3].

The common English names are: arbutus, cane apples, Irish strawberry tree, Killarney strawberry tree, strawberry madrone, and strawberry tree. The vernacular names in several southern European countries are: ervedeiro, medronheiro (Portugal); albocera, alborocera, borrachín, madroñera, madroño (Spain); arbousier, arbousier commun, fraisier en arbre (France); corbezzolo, sorbo peloso (Italy); and koumaria (Greece) [3].

*A. unedo* grows to 9–12 m tall, but is normally between 1.5 m to 3 m tall [4]. The bark is fissured and it peels off in small flakes, mostly dull brown. The leaves are alternate, simple, oblanceolate, dark green, leathery and have a serrated margin, usually 2–3 times as long as wide, glabrous with a petiole of 10 mm or less [5,6]. The flowers, with recurved lobes, are bell-shaped, 8–9 mm long, white, and often pale pink [5]. *A. unedo* flowers are a significant source of nectar and pollen for bees [6]. The fruits are globular, orange-red when ripe, growing up to 2 cm in diameter, are recovered with conical papillae and mature in autumn [2,6]. Fruits take about 12 months to ripen; therefore, the tree carries mature fruits and flowers at the same time. The flowering and fructification process extends from October to February [7].

*A. unedo* prefers siliceous or decarbonated substrata and can grow on alkaline and relatively acidic soils (pH 5–7.2) [1,4,6,8]. This species has a huge ecological importance since it prevents erosion of the soils and has also the capacity to regenerate itself rapidly after fires, surviving quite well in poor soils [7,9]. Strawberry trees are characterized by a wide genetic, morphological and phenological variability [4,6,7,10,11,12]. Some studies have demonstrated that drought delays flowering and fruit development in *A. unedo*. Summer drought and low minimum temperatures during the flowering phase reduce fruit yields [13,14].

An ethnobotanical study, describing the wild edible plants in Biscay (Spain), found that strawberry tree fruits had a significant cultural importance in that region, along with other nineteen wild edible plants [15]. Nevertheless, fruits of *A. unedo* are rarely eaten as fresh fruit as despite their pleasing appearance, the taste is not much appreciated by the consumer. So far, they are generally used for obtaining alcoholic drinks (wines, liqueurs and brandies), jams, jellies and marmalades. They can also be incorporated into yoghurts either in pieces or as flavours and be used, like other berries in confectionery such as pie and pastry fillings, cereal products, among other applications [16,17,18].

In traditional folk medicine, *A. unedo* has been used in antiseptics, diuretics and laxatives and to treat arterial hypertension. The leaves have been reported as possessing several biological properties such as astringent, human platelet anti-aggregant due to its relative high amounts of tannins, urinary antiseptic, anti-inflammatory, anti-diarrheal, anti-hypertension and anti-diabetic [19,20,21,22,23,24,25,26,27,28,29,30,31,32]. The present work is focused in the chemical composition of different parts of the *A. unedo* shrub and its derived products (honey and spirit beverages) as well as their biological properties.

## 2. Chemical Composition of *A. unedo*

### 2.1. Fruits

#### 2.1.1. Phenols

**Figure 1:**
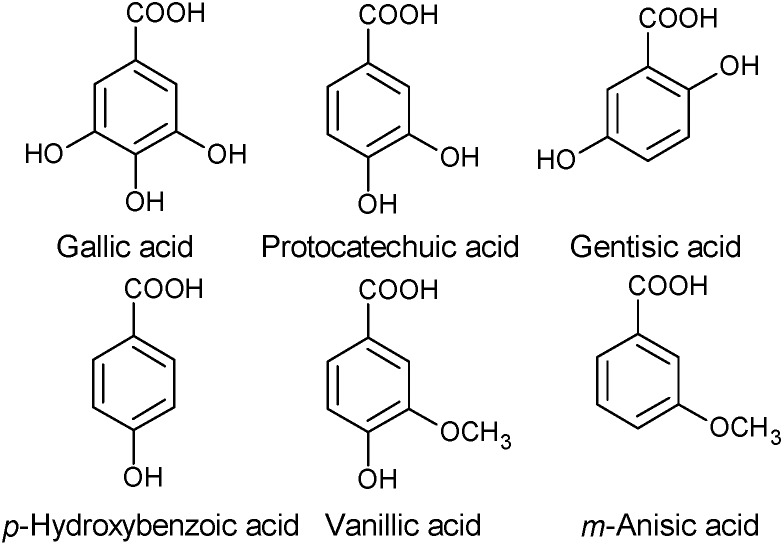
Some simple phenolics found in *A. unedo* fruits collected in Samsun (Turkey).

Several components belonging to diverse phenol groups have been reported in *Arbutus* fruits: phenolic acids, flavonols, flavan-3-ols and galloyl derivatives, and anthocyanins [16,21,22,26,27,33,34,35]. Gallic acid (10.7 mg/g, dry weight) was the main phenolic compound reported by [16] in *A. unedo* fruits collected in Samsun (Turkey), followed by protocatechuic acid, gentisic acid, *p*-hydroxybenzoic acid, vanillic acid and *m*-anisic acid (Figure 1).

After separation by HPLC and on the basis of spectral identification, phenolics of full ripeness strawberry tree fruits from the Cáceres region (Spain) were divided into seven subclasses and quantified: catechin and procyanidin (expressed as (+)-catechin equivalents; detection wavelength, 280 nm); hydroxybenzoic acids (as gallic acid equivalents, 280 nm); ellagitannins (as ellagic acid equivalents, 280 nm); ellagic acid (as ellagic acid equivalents, 365 nm); hydroxycinnamic acids (as cholorogenic acid equivalents, 320 nm), flavonols (as rutin equivalents, 365 nm); and anthocyanins (as cyanidin-3-glucoside equivalents, 520 nm), expressed as mg/100 g (d.w.). The concentrations found by the authors were: catechins (313.4); hydroxybenzoic acids (112.2); hydroxycinnamic acids (1.0); flavonols (3.6); ellagic acid (6.9); anthocyanins (5.8); procyanidins (474.1) [33].

**Figure 2:**
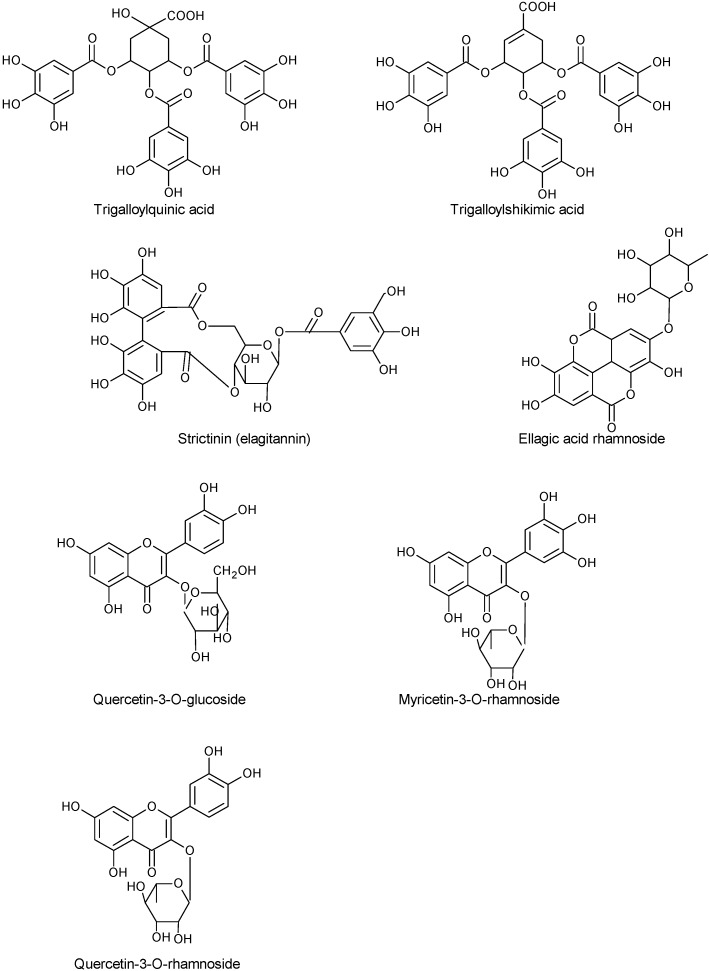
Some chemical structures of galloyl derivatives, tannins and flavonols present in *A. unedo* fruits.

The main phenolic compounds in wild fruits from North-eastern Portugal were flavan-3-ols and galloyl derivatives (60.93 mg/100 g), followed by anthocyanins (13.77 mg/100 g) and flavonols (10.86 mg/100 g) [34]. Identification of phenolic compounds was done by means of liquid chromatography (HPLC) coupled to a diode array detector (DAD) and mass spectrometry (MS) using the electrospray ionization interface (ESI). Quercetin galloylhexoside derivatives, quercetin-3-*O*-rutinoside, quercetin-3-*O*-glucoside, quercetin pentoside, quercetin rhamnoside, kaempferol hexoside, and myricetin rhamnoside constitute the flavonols found by the authors in *Arbutus* fruits. Within the group of flavan-3-ols (36.30 mg/100 g) and galloyl derivatives (24.63 mg/100 g), the fruits had B_1_ dimer, B-type proanthocyanidin trimers, B-type procyanidin tetramer, B-type procyanidin dimer, galloylquinic acid, galloylhexoside acid, galloylshiquimic acid, (+)-catechin, digalloylquinic acid, digalloylquinic shikimic acid and strictinin ellagitannin. Delphinidin-3-*O*-glucoside, cyanidin-3-*O*-glucoside and cyanidin-3-*O*-pentoside were the anthocyanins identified and quantified by the authors in *Arbutus* fruits from Northeastern Portugal [34]. Previously, [27] had already identified by HPLC-DAD/ESI-MS, several phenolic compounds in fruits of *A. unedo* collected in the Natural Park of Montesinho (Bragança, Northeast of Portugal): gallic acid glucoside, galloylquinic acid, quinic acid derivative, proanthocyanidin dimer, galloylshikimic acid, digalloylquinic acid, digalloylshikimic acid, catechin monomer, proanthocyanidin trimer, strictinin ellagitannin, ellagitannin derivative, galloyl derivative, trigalloylshikimic acid, myricetin rhamnoside, quercetin glucoside, gallotannin, ellagic acid rhamnoside (Figure 2). Practically the same phenolics and their derivatives were also identified in the extracts of arbutus fruits collected in Arrábida Natural Park (Southern region of Portugal) [26].

The phenolic composition of fruits of strawberry tree from Salamanca, western Spain, was evaluated in [22]. Some anthocyanins found by these authors in fruits were different from those found in Portugal. Spanish fruits of strawberry fruits had delphinidin-3-galactoside, cyanidin-galactoside, cyanidin-glucoside, and cyanidin-arabinoside. Among these anthocyanins, cyanidin-galactoside prevailed (2.8 mg/100 g edible portion). The main anthocyanin in fruits from Portugal was cyanidin-3-*O*-glucoside (11.40 µg/100 g, dry weight). Beyond the qualitative differences between fruits from Portugal and Spain, quantitative differences can also be detected. Such differences may be attributed to the different ways of quantification, among other reasons not discussed in the present work. In Portuguese fruits, the authors [34] used an external standard for every anthocyanin, whereas in Spanish fruits, the authors [22] used only one calibration curve obtained with cyanidin-3-glucoside.

**Figure 3:**
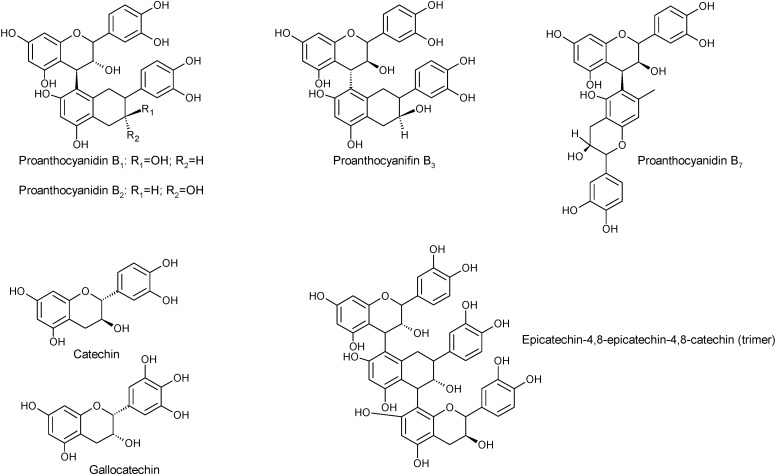
Chemical structures of the most abundant proanthocyanidins present in *A. unedo* fruits.

In the flavonol group there were also some differences between fruits from Portugal and Spain. In this country, the authors [22] reported myricetin-3-*O*-xyloside, quercetin-3-*O*-xyloside (both not reported in Portuguese fruits), quercetin-3-*O*-rutinoside and quercetin-3-*O*-rhamnoside. Within the group of flavan-3-ols and galloyl derivatives, [22] reported as being present in fruits of strawberry tree, gallocatechin, gallocatechin-4,8-catechin, the proanthocyanidins B_1_ (epicatechin-4,8-catechin and epicatechin-4,6-catechin), B_2_ (epicatechin-4,8-epicatechin), B_3_ (epicatechin-4,8-catechin), and B_7_ (epicatechin-4,6-catechin) dimers, epicatechin, epicatechin-4,8-epicatechin-4,8-catechin (Figure 3) and epicatechin-4,8- epicatechin-4,8-epicatechin.

Extracts of strawberry tree fruits, after enzymatic hydrolysis with hesperidinase followed by cellulose, had gallic acid, protocatechuic acid, (+)-catechin, phloroglucinaldehyde, ellagic acid, myricetin and quercetin [35]. The conjugated sugars found by these authors in the extracts were glucoside, galactoside, rutinoside, rhamnoside and arabinoside.

**Figure 4:**
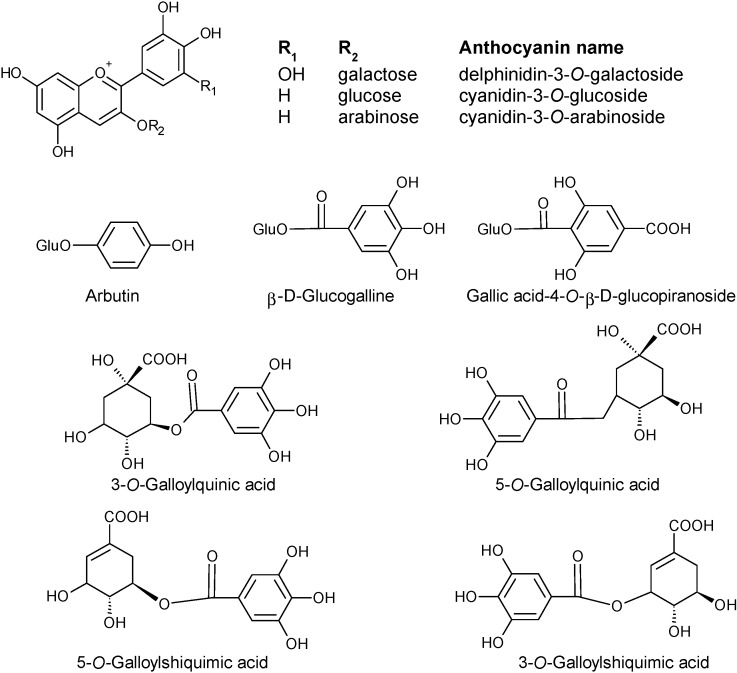
Chemical structures of some phenolic compounds, including anthocyanins, of *A. unedo* fruits collected in Italy.

The phenolic constituents of *A. unedo* fruits collected in Marina di Vecchiano, Pisa, Italy, included anthocyanins (delphinidin-3-*O*-galactoside, cyanidin-3-*O*-glucose and cyanidin-3-*O*-arabinoside); 4-arbutin, β-d-glucogalline; 3-*O*-galloylquinic acid; gallic acid-4-*O*-β-d-glucopyranoside; 5-*O*-galloylquinic acid; 5-*O*-galloylshikimic acid; and 3-*O*-galloylshikimic acid (Figure 4), identified by HPLC-DAD-ESI-MS, ^1^H- and ^13^C-NMR [21].

#### 2.1.2. Fatty Acids

α-Linolenic (36.51%), linoleic (21.50%) and oleic acids (21.01%) were the predominant unsaturated fatty acids, and palmitic acid the most important saturated fatty acid (8.20%) found by [36] in ripe fruits of strawberry tree fruits collected in the Natural Park of Montesinho territory (Trás-os-Montes, north-eastern Portugal). Fatty acids composition checked in fruits at different ripening stages and collected in Trás-os-Montes (North-eastern Portugal) showed that their profiles were very similar between the unripe and ripe stages, being α-linolenic (36.9%–43.04%, respectively), linoleic (20.14%–18.84%, respectively) and oleic acids (29.38%–26.75%, respectively), the three major ones. α-Linolenic (31.26%), linoleic (24.26%) and oleic acids (24.82%) were also predominant in fruits collected in central and western Spain [37]. Polyunsaturated fatty acids (PUFA) were the major fraction of fatty acids either in Portuguese or Spanish fruits, represent at least 52% of the total fatty acids. These fruits had a highly favourable ω3/ω6 ratio, due to the richness in α-linolenic [29], as well as a good PUFA/SFA (saturated fatty acids) (2.85), considered as cardioprotective [36,37].

Strawberry tree fruits from Croatia also had as main fatty acids linoleic and linolenic acids (34.8% and 31.3%, respectively), but the third more important was palmitic acid (19.0%) and not oleic acid (14.9%) [38] as reported above for the other samples from different locations.

#### 2.1.3. Vitamins and Others

**Figure 5:**
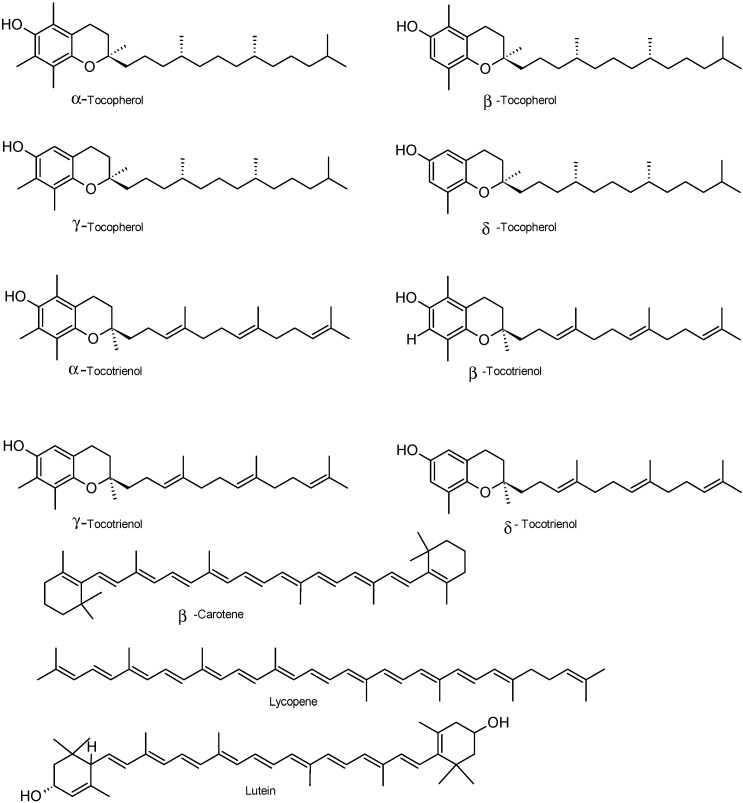
Chemical structure of vitamin E and some carotenoids of *A. unedo* fruits.

Fruits of strawberry tree from Trás-os-Montes (North-eastern Portugal) contained vitamin E. Among the vitamin E vitamers, the most important was γ-tocotrienol. The total free vitamin E content reduced with ripening: from 1369 mg/kg in unripe fruits to 557 mg/kg in ripe ones [29]. α-, β-, γ- and δ-Tocopherols and α-tocotrienol were also present in fruits (Figure 5). Other studies characterizing the nutrients and phytochemicals with antioxidant properties of strawberry tree fruits collected in the Natural Park of Montesinho territory, in Trás-os-Montes, North-eastern Portugal, also showed the presence of tocopherols (235 mg/kg), being α-tocopherol the most important (235 mg/kg), followed by γ- tocopherol and β-tocopherol [36]. These authors did not find δ-tocopherol in fruits.

The presence of α-tocopherol was also reported by [22], nevertheless in much lower amounts (0.2 mg/kg) than those reported by other authors [29,36] (271 mg/kg in unripe fruits to 32 mg/kg in ripe fruits). Total vitamin E in wild fruits of strawberry fruits from Central and Western Spain was also evaluated by [37]. The concentration (39 mg/kg) found by these authors were similar to those reported by [29] in ripe fruits. Among the vitamin E isomers, the authors found that α-tocopherol predominated (35 mg/kg), followed by γ- tocopherol, β-tocopherol and δ-tocopherol.

Vitamin C has also been reported as being present in arbutus fruits but found in distinct concentrations: 89 mg/100 g [35]; from 542 mg/100, in unripe fruits, to 346 mg/100 g, in red mature fruits [17]; 6 mg/100 g [22]; 119.1 mg/100 g [39]; and 182 mg/100 g [37]. Studies performed by [31] demonstrated the importance of harvest date and location on the amounts of total vitamin C (262.7–122.0 mg/100 g, f.w.); ascorbic acid (203.8–93.1 mg/100 g); dehydroascorbic acid (90.8 mg/100 g—not detected); β-carotene (0.808–0.243 mg/100 g); and lycopene (0.209 mg/100 g—not detected).

β-Carotene, niacin, lutein+zeaxanthine, and β-cryptoxanthyne were also reported as constituents of Spanish and Portuguese fruits of strawberry tree [17,22]. Ripe fruits collected in Croatia contained 271 mg/100 g (f.w.) vitamin C, of which 255.3 mg/100 g was ascorbic acid and 16.2 mg/100 g was dehydroascorbic acid [38]. In North-western Turkey, wild strawberry tree fruits had ascorbic acid (270.50 mg/100 g) [40], which concentration is within the range found for other samples with different origins [31,38].

**Figure 6:**
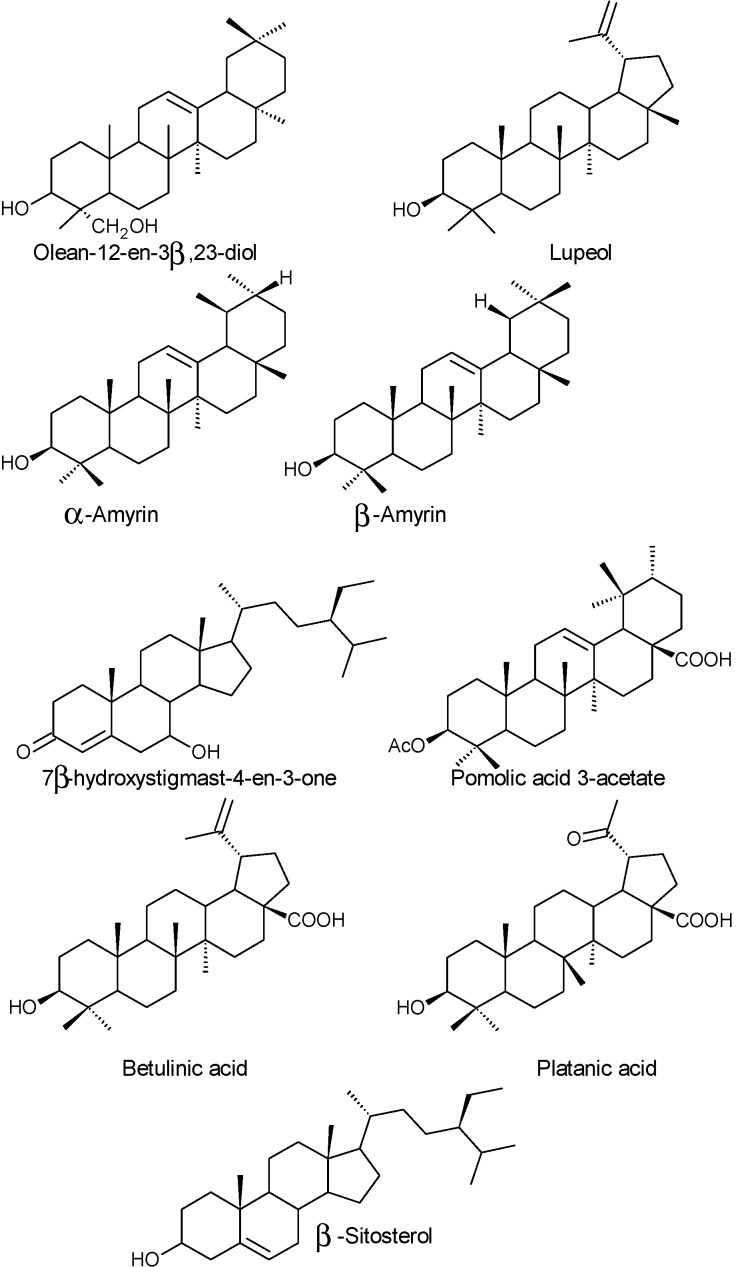
Chemical structure of some triterpenes found in fruits and other parts of *A. unedo*.

Triterpenes were also found in fruits of *A. unedo* when separated and identified by high pressure liquid chromatography coupled to a mass spectrometer by means of a particle beam interface (HPLC-PBMS). Through this methodology, the authors identified α- and β-amyrin, lupeol as well as a new natural triterpene (olean-12-en-3β,23-diol) (Figure 6) [41].

#### 2.1.4. Organic Acids

**Figure 7:**
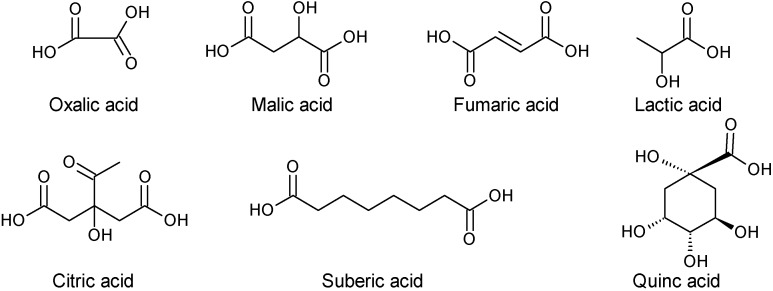
Chemical structures of some organic acids found in *A. unedo* L.

Fumaric (1.49 mg/g, d.w.), lactic (0.49 mg/g), malic (0.84 mg/g), suberic (0.23 mg/g) and citric (0.01 mg/g) acids were the organic acids detected and quantified by [16] in *A. unedo* fruits from Samsun, Turkey. In Spain, fruits of strawberry tree had only oxalic acid (96.53 mg/100 g, f.w.); and fumaric acid (0.73 mg/100 g), according to the results reported by [37]. Some variability in the organic acids content of strawberry tree fruits was found in samples gathered in three different seasons (November and December 2007–2009) and from two localities with different environmental conditions (Madrid, center of Spain; and Cáceres, west of Spain): oxalic acid (146.75–48.44 mg/g, f.w.); malic acid (314.94–203.3 mg/g); and fumaric acid (0.919–0.539 mg/g) [31]. In Portugal, fruits harvested in Tapada da Ajuda, Lisboa, had malic and quinic acids (Figure 7), which concentrations depended on the ripening stage of fruits [17]. In contrast to those samples from Turkey, in which fumaric acid predominated, in Portugal only traces were detected mainly in red mature fruits.

#### 2.1.5. Sugars

Fructose (27.8%, d.w.) and glucose (21.5%, d.w.) were the predominant sugars in fruits from Turkey, followed by sucrose (1.80%) and maltose (1.11%) [16]. Similar concentrations of fructose, glucose and sucrose were also reported by Şeker and Toplu [40] in *Arbutus* fruits collected in North-western Turkey (24.09, 19.09% and 2.65%, d.w., respectively). In the Natural Park of Montesinho territory, in the Trás-os-Montes, North-eastern Portugal, the major sugar was also fructose (24.21%, d.w.) in fruits of strawberry tree, but the glucose amount was much lower (12.14%) and sucrose level was much higher (4.20%) than those reported by [16,40] for Turkish fruits. Alarcão-e-Silva *et al.* [17] found that the amounts of glucose and fructose were dependent on the ripening stage, whereas sucrose content did not change (8.77 and 8.68%, d.w.) in unripe and red mature fruits, respectively. Glucose amount ranged from 3.95 in unripe fruits to 12.5% in red mature fruits (d.w.). This difference between two ripening stages was even higher for fructose (2.33–20.8, d.w., respectively). A great variability in fructose (12.69%–3.65%, f.w.) and glucose (6.50%–2.34%) amounts were reported by [31] in fruits of *A. unedo*, depending on the harvest date and location of the orchards (center and west of Spain). For the same samples, the levels of sucrose ranged from 0.48% to absence. The levels of fructose and glucose in strawberry tree fruits collected in Croatia were within the range reported for Portuguese and Spanish samples (12.62% and 5.27%, respectively) [38].

#### 2.1.6. Volatiles

The analysis of the volatiles of strawberry tree fruits performed using headspace-solid phase micro-extraction (HS-SPME) allowed the identification of a total of 41 compounds in Portuguese samples. The fruits were collected at different ripening stages in the region of Trás-os-Montes (north-eastern Portugal). Alcohols [(*Z*)-3-hexen-1-ol, 1-hexanol] are the main volatile compounds detected in the three ripening stages, followed by aldehydes [hexanal, (*E*)-2-hexenal], and esters [(*Z*)-3-hexenyl acetate] [42]. Generally, these volatiles decreased during ripening, maybe due to the reduction of the activity of lipoxygenase [42]. The remaining chemical classes found in fruits were norisoprenoid derivatives, sesquiterpenes and monoterpenes but in very low amounts. In the progress of the fruit ripening, the amounts of monoterpenes decrease from unripe to intermediate maturation, presenting in the ripe stage the highest quantity; whereas sesquiterpenes increase their amount from unripe to intermediate fruits, after which their presence was lower. Norisoprenoids derivatives decrease their presence as the maturation increases [42].

The essential oil isolated from fruits of *A. unedo* in the north-eastern Turkey by hydrodistillation and analysed by GC-MS was mainly constituted by hexadecanoic acid, ethyl dodecanoate, ethyl linoleate, tetradecanoic acid, and *trans*-carane [43]. Flower oil had a different chemical profile because the major components were hexadecanoic acid, α-terpineol, nonanal and linalool [43].

#### 2.1.7. Minerals

The mineral composition of arbutus fruits reported for Turkish samples collected in Mersin (Turkey) was: Ca (4959 mg/kg) , K (14,909 mg/kg), Mg (1316 mg/kg) , Na (701 mg/kg) and P (3669 mg/kg) as main minerals, whereas Cd, Cu, Li Mn, Ni, Pb and Sr contents were very low [44]. In other work, fruits collected in the North-eastern Anatolia region of Turkey had K (119 mg/100 g); P (12.6 mg/100 g); Ca (12 mg/100 g); Mg (9.1 mg/100 g); Fe (1.25 mg/100 g); Cu (0.088 mg/100 g); Zn (2.602 mg/100 g); and Mn (0.197 mg/100 g) [39]. In the North-western regions of Turkey, K (11,346 mg/kg and 13,661.45 mg/kg); Ca (5673.39 mg/kg and 5475.62 mg/kg); P (4278.38 mg/kg and 4554.91 mg/kg); Mg (1691.46 mg/kg and 1931.49 mg/kg); and Na (1136.79 mg/kg and 1034.53 mg/kg) were also the main minerals found by [40,45]. Samples collected in Croatia had K (118.61 mg/100 g); Na (20.63 mg/100 g); Ca (36.05 mg/100 g); Mg (9.66 mg/100 g); Fe (1.29 mg/100 g); P (19.99 mg/100 g); Zn (0.45 mg/100 g); Mn (<0.99 mg/100 g); Cr (<0.99 mg/100 g); Ni (<0.10 mg/100 g); Pb (<1.32 mg/100 g); and Cd (<0.10 mg/100 g) [38].

A wide variability in the mineral composition of strawberry tree fruits was found in samples gathered in three different seasons (November and December 2007–2009) and from two localities with different environmental conditions (Madrid, center of Spain; and Cáceres, west of Spain): Na (9.94–4.33 mg/100 g, f.w.); K (323.14–79.72 mg/100 g); Ca (104.12–40.54 mg/100 g); Mg (45.85–9.56 mg/100 g); Cu (0.208–0.073 mg/100 g); Fe (1.856–0.354 mg/100 g); Mn (0.178–0.038 mg/100 g); and Zn (0.762–0.188 mg/100 g) [31]. According to the results of the authors, the year of harvest significantly influenced the fruit content of K, Mg, Cu, Fe, Mn and Zn.

### 2.2. Leaves

Arbutin was reported as being present in leaf extracts of *A. unedo* collected in Montenegro, along with hydroquinone derivatives [46]. Quercitrin, isoquercitrin, hyperoside, and chlorogenic acid are other phenolic compounds identified and quantified by [5,47] in extracts of leaves of *A. unedo* from Croatia. In this work, the authors also reported that the concentrations of these compounds changed over the year. For example, higher concentrations of hyperoside and quecitrin were found in January, whereas chlorogenic acid was in higher amounts in June, July and October. 

Leaf extracts of *A. unedo* from the Natural Park of Montesinho (Bragança, Northeast of Portugal) after LC-DAD-ESI/MS were shown to have flavanols (catechins, procyanidin dimers and respective gallate esters), flavonols (glucosides of myricetin, quercetin, kampferol), several galloyl (gallotannins) and ellagic (ellagitannins) derivatives. These compounds were also present in fruit extracts; however the number of compounds identified was higher in leaf extracts than in fruit [27].

α-Tocopherol was also found in leaves of *A. unedo* from Turkey, being the amounts found dependent on the collection season. Leaves collected in March had the highest concentration of α-tocopherol [48].

The essential oil of *A. unedo* leaves, collected in West Anatolia in Izmir-Cicekliköy (Turkey), obtained by hydrodistillation had (*E*)-2-decenal, α-terpineol, hexadecanoic acid, and (*E*)-2-undecenal as main components [49]. A distinct chemical composition was reported for essential oil of leaves isolated from *A. unedo* of Algerian origin: palmitic acid, linoleic acid and 2,6-di-tert-butyl-*p*-cresol [50]. Volatile organic compounds emitted by the aerial parts of *A. unedo*, including leaves, were also studied in fields of Italy and Spain. Nonanal, decanal, 2-ethoxyethyl acetate and monoterpene hydrocarbons and oxygenated monoterpene were identified [51,52].

### 2.3. Stems

Catechin and epicatechin are monomers constituting the proanthocyanidin also known as condensed tannins, a broad family of oligomers and polymers belonging to the flavonoid class. The stems of *A. unedo* L. from Algeria after extraction using a water/methanol/acetone mixture and structural analysis by ^1^H-NMR, ^13^C-NMR, IR and mass spectra using an ion-trap spectrometer, operating on an ESI mode showed the presence of (+)-catechin, (+)-afzelechin and (3,4-dihydroxyphenyl)-5,7-dihydroxychroman-3-yl 4-hydroxybenzoate [53].

### 2.4. Roots

The roots of *Arbutus unedo* L. from the Terni forest (Tlemcen, Algeria) extracted with a water/methanol/acetone mixture had two major compounds identified by NMR spectroscopy: (+)-catechin and (+) catechin gallate. Other phenolic compounds were also identified by GC-MS such as benzoic acid, 4-(acetyloxy)-3-methoxy-, methyl ester; 4-hydroxyphenyl acetic acid; caffeic acid; gallic acid; protocatechic acid and *bis*(2-ethylhexyl) phthalate [54].

### 2.5. Seeds

The triacylglycerol characterisation of *A. unedo* seeds made by [55] revealed low saturated fatty acids, high content of oleic acid, a significant presence of ω6 and ω3 unsaturated fatty acids with a low ω6/ω3 fatty acid ratio. The distribution of fatty acids among the three *sn*-positions of triacylglycerol is asymmetric, nevertheless with a high incorporation of essential fatty acids in the *sn*-2-position, which is very important from a nutritional point of view [55].

### 2.6. Entire Plant

From the hydro-alcoholic extract of the entire plant of *A. unedo* collected in a Mediterranean woodland coppice, located in Central Italy, 12 phenolic compounds were identified: ethyl gallate, arbutin and two arbutin derivatives (*p*-hydroxybenzoyl arbutin and galloyl arbutin) and eight flavonoids (gallocatechin, catechin, kaempferol 3-*O*-α-l-rhamnoside, quercetin 3-*O*-α-l-rhamnoside, myricetin 3-*O*-α-l-rhamnoside, kaempferol 3-*O*-β-d-arabinoside, quercetin 3-*O*-β-d-arabinoside, and myricetin 3-*O*-β-d-arabinoside) [56].

Triterpenes were also found in the petroleum ether and ethyl acetate extracts of entire plant collected in Turkey: α-amyrin acetate, betulin, betulinic acid, 6β-hydroxystigmast-4-en-3-one, lupeol, platonic acid, pomolic acid 3-acetate, β-sitosterol and 7β-hydroxystigmast-4-en-3-one (Figure 6) [57].

## 3. Biological Properties and Applications

### 3.1. Fruits

The extracts of *A. unedo* fruits revealed to have *in vitro* antioxidant activity [26,29,36,37,40,58,59,60,61,62]. The type of extraction of phenols present in fruits of *A. unedo* influenced the antioxidant activity. The capacity for scavenging free radicals of the ripe fruit extracts of strawberry tree obtained by supercritical fluid extraction in the adequate values of pressure (60 bar), temperature (48 °C), concentration of co-solvent (ethanol 19.7%) by CO_2_ flow rate of 15 g/min for 60 min, at a solvent/feed ratio of 30 was better when compared to that obtained by solid-liquid extraction (Soxhlet) using ethanol as extraction solvent, but similar when compared to the water extracts. In contrast, the capacity for preventing lipid peroxidation, measured through the β-carotene bleaching method, was better in water extract [58]. According to these authors this extract had the lowest oxidation rate (0.661) and the highest activity coefficient (809) in comparison to supercritical CO_2_ (0.958 and 492, respectively) and ethanol extracts (1.101 and 330, respectively).

The antioxidant capacity of fruit extracts reported by [59] was higher when obtained from fully red fruits, except H_2_O_2_ scavenging activity which was higher in green fruit. The concentrations of red and yellow fruit extracts providing 50% antioxidant activity (EC_50_) were 0.409 and 0.499 mg/mL, respectively, in the DPPH (2,2-diphenyl-1-picrylhydrazyl) method. The EC_50_ values found when the β-carotene bleaching method was used were 0.246 and 0.328 mg/mL in red and yellow fruit extracts, respectively. The H_2_O_2_ scavenging capacity was low (27.10% and 25.86% for green and red fruit extracts, respectively). The influence of maturation stage on antioxidant activity measured through the DPPH method was also found by [29]. Intermediate (EC_50_ = 0.37 mg/mL) and ripe fruits (EC_50_ = 0.25 mg/mL) of strawberry tree fruits possessed higher antioxidant activity than unripe fruits (EC_50_ = 0.58 mg/mL). Storage of fresh fruits of *A. unedo* at 0 °C preserved better the antioxidant activity than at higher temperatures (3 and 6 °C) during the experimental period (15 days) [61], evaluated by the capacity for scavenging peroxyl radicals [Oxygen Radical Absorbance Capacity—ORAC method] and 2,2'-azino-bis(3-ethylbenzothiazoline-6-sulphonic acid) (ABTS) cation radicals [Trolox Equivalent Antioxidant Capacity-TEAC method]. In the same experiment, the authors also reported that the type of films covering the fruits (linear low density polyethylene or polyethylene film perforated with holes) did not influence the antioxidant activity of fresh fruits [61].

The antioxidant activity was influenced by the drying fruit processes. Some authors [60] reported that fruits submitted to freeze drying had higher capacity for scavenging DPPH free radicals (EC_50_ = 2.125 mg/mL) and preventing lipid peroxidation (EC_50_ = 0.290 mg/mL) than those submitted to hot air drying (EC_50_ = 6.956 and 0.880 mg/mL, respectively). This loss when using hot air drying would be expectable due to the possible degradation and/or loss of some compounds such as phenols and vitamins [60,63].

Beyond the antioxidant activity of fruit extract of *A. unedo* found by [62] measured through the capacity of the extract with phenolic acids, flavones/ols, flavan-3-ols and galloyl derivatives to inhibit peroxidation in animal brain homogenates, the same extract had also antitumor potential against NCI-H460 human cell line (non-small lung cancer). The authors obtained two main types of extracts from *Arbutus unedo*, *Prunus spinosa*, *Rosa micrantha* and *Rosa canina* fruits collected in Portugal: non-anthocyanin phenolic compounds enriched extract and anthocyanins enriched extract. Non-anthocyanin phenolic compounds enriched extract of *A. unedo* had the highest capacity to inhibit lipid peroxidation in animal brain homogenates (EC_50_ = 7.21 μg/mL) as well as a high antitumor potential against NCI-H460 human cell line. The authors considered that such activities might be attributed to the presence of galloyl derivatives exclusively found in this species [62].

The antioxidant ability of strawberry tree fruits has led to the application of their phenolic-rich extracts as functional ingredients in processed meat products [33,64,65,66]. For example, the combination of fruits extracts together with sodium ascorbate and sodium nitrite enhance the oxidative stability of frankfurters without modifying their colour and texture properties. The utilization of fruit extracts also minimized the deterioration of quality during the refrigerated storage [64]. The addition of extracts from *A. unedo* fruits in porcine burger patties protected polyunsaturated fatty acids from oxidative degradation, inhibited the formation of thiobarbituric acid reactive substances and volatiles compounds; and the oxidation of proteins preventing the formation of carbonyl compounds [33,65,66]. The utilization of these extracts as antioxidants enhanced the nutritional, safety and sensory properties of porcine burger patties [33,65,66].

Other applications of fruit extracts in food technology have been reported, such as the enrichment of yogurts with fruit extract of *A. unedo* which improved the antioxidant activity and the survival of its microbial community, not affecting the chemical and microbiological characteristics of yogurts [67]. Fruit essential oil of *A. unedo* showed a moderate antibacterial activity against *Listeria monocitogenes* and *Enterococcus faecalis* [43].

### 3.2. Leaves

Antioxidant activity in chemical based model assays has been reported for leaf extracts of *A. unedo* [20,28,30,32,68,69], and cell based model assays [27]; antibacterial activity, mainly against Gram-positive bacteria [68,69,70], *Helicobacter pylori* and *Klebsiella pneumoniae* [71]; antifungal effect against two aflatoxigenic molds namely *Aspergillus parasiticus* NRRL 2999 and NRRL 465 [68] and against *Candida tropicalis* [71]; intracellular anti-mycobacterial activity without toxic effect on macrophages, especially ethanolic extract [70] (Table 1); *in vitro* anti-leishmanial activity, mainly ethanolic extracts [72]; *in vitro* activity against *Trichomonas vaginalis* trophozoites, mainly an ethyl acetate extract [73]; anti-inflammatory activity of aqueous extracts by down-regulating Signal Transducer and Activator of Transcription 3 (STAT3) activation induced by injection of carrageenan in the pleural cavity of mice [74], or by down-regulation of STAT1 elicited by IFN-γ (interferon-γ), both in human breast cancer cell line MDA-MB-231 and in human fibroblasts [75].

**Table 1:** Antimicrobial activities reported for leaf extracts of *A. unedo.*

Microorganism	Extract	Activity		Ref.
Gram Positive				
*Staphylococcus aureus*	Aqueous	Inhibition zone diameter	10.5 mm (10 μL/disc)	[68]
			13.8 mm (20 μL/disc)	
			21.1 mm (40 μL/disc)	
*Bacillus cereus*	Aqueous	Minimum inhibitory concentration (MIC)	1 mg/mL	[69]
*B. subtilis*			1 mg/mL	
*Staphylococcus epidermis*			1 mg/mL	
*S. aureus*			2.5 mg/mL	
*B. cereus* ATCC 11771	Acetone/water (60:40)	Inhibition zone diameter	10.0 mm (2 mg/disc)	[71]
	Ethanol 95%		11.3 mm	
	Methanol (Soxhlet)		10.0 mm	
	Methanol (maceration)		10.7 mm	
	Ethanol (maceration)		9.3 mm	
*S. aureus* ATCC 25923	Acetone/water (60:40)	Inhibition zone diameter	9.3 mm	[71]
	Ethanol 95%		12.0 mm	
	Methanol (Soxhlet)		10.3 mm	
	Methanol (maceration)		9.3 mm	
	Ethanol (maceration)		9.3 mm	
*Enterococcus faecalis* ATCC 29212	Acetone/water (60:40)	Inhibition zone diameter	8.0 mm	[71]
	Ethanol 95%		6.5 mm	
	Methanol (Soxhlet)		7.0 mm	
	Methanol (maceration)		7.3 mm	
	Ethanol (maceration)		7.3 mm	
Clinical Methicillin-Resistant *Staphylococcus aureus* 10/8	Acetone/water (60:40)	Inhibition zone diameter	7.8 mm	[71]
	Ethanol 95%		8.8 mm	
	Methanol (Soxhlet)		--	
	Methanol (maceration)		8.0 mm	
	Ethanol (maceration)		8.0 mm	
Clinical Methicillin-Resistant *Staphylococcus aureus* 12/8	Acetone/water (60:40)	Inhibition zone diameter	7.0 mm	[71]
	Ethanol 95%		9.5 mm	
	Methanol (Soxhlet)		--	
	Methanol (maceration)		--	
	Ethanol (maceration)		--	
*Mycobacterium smegmatis*	Aqueous	MIC	6.02 mg/mL	[70]
*M. aurum A+*			5.59 mg/mL	
*M. bovis PPI*			6.02 mg/mL	
Gram negative				
*Pseudomonas aeruginosa*	Aqueous	MIC	5 mg/mL	[69]
*Escherichia coli*			5 mg/mL	
*Klebsiella pneumoniae* ATCC 13833	Acetone/water (60:40)	Inhibition zone diameter	9.0 mm (2 mg/disc)	[71]
	Ethanol 95%		10.8 mm	
	Methanol (Soxhlet)		9.8 mm	
	Methanol (maceration)		9.3 mm	
	Ethanol (maceration)		8.7 mm	
*Helicobacter pylori* 104/98	Acetone/water (60:40)	Inhibition zone diameter	18.3 mm	[71]
	Ethanol 95%		14.6 mm	
	Methanol (Soxhlet)		17.7 mm	
	Methanol (maceration)		16.7 mm	
	Ethanol (maceration)		13.5 mm	
*Helicobacter pylori* P10/92	Acetone/water (60:40)	Inhibition zone diameter	17.8 mm	[71]
	Ethanol 95%		14.6 mm	
	Methanol (Soxhlet)		15.5 mm	
	Methanol (maceration)		14.6 mm	
	Ethanol (maceration)		14.9 mm	
*Helicobacter pylori* 93/00	Acetone/water (60:40)	Inhibition zone diameter	12.9 mm	[71]
	Ethanol 95%		11.1 mm	
	Methanol (Soxhlet)		12.3 mm	
	Methanol (maceration)		11.8 mm	
	Ethanol (maceration)		11.5 mm	
*Helicobacter pylori* B22/96	Acetone/water (60:40)	Inhibition zone diameter	18.0 mm	[71]
	Ethanol 95%		17.5 mm	
	Methanol (Soxhlet)		18.2 mm	
	Methanol (maceration)		18.3 mm	
	Ethanol (maceration)		15.8 mm	
*Helicobacter pylori* ATCC 710392	Acetone/water (60:40)	Inhibition zone diameter	10.4 mm	[71]
	Ethanol 95%		7.6 mm	
	Methanol (Soxhlet)		9.1 mm	
	Methanol (maceration)		9.0 mm	
	Ethanol (maceration)		8.1 mm	
Fungi				
*Aspergillus parasiticus* NRRL 2999 (0.075%–0.3%)	Aqueous	Inhibitory effect	30.15%–42.70%	[68]
*Aspergillus parasiticus* NRRL 465 (0.075%–0.3%)			12.79%–29.76%	
Yeasts				
*Candida tropicalis* ATCC 750	Acetone/water (60:40)	Inhibition zone diameter	9.3 mm (2 mg/disc)	[71]
	Ethanol 95%		13.3 mm	
	Methanol (Soxhlet)		10.7 mm	
	Methanol (maceration)		12.0 mm	
	Ethanol (maceration)		12.7 mm	

The antioxidant activity was dependent on the solvent extraction [20,28,68]. The Trolox equivalent antioxidant capacity of ethanol and methanol extracts of *A. unedo* leaves were 2.25 mM and 1.75 mM, respectively [20]. Ethanol extracts of *A. unedo* leaves were the highest in reducing power (IC_50_ = 232.7 μg/mL) and DPPH scavenging effect (IC_50_ = 63.2 μg/mL), followed by water extracts (IC_50_ = 287.7 and 73.7 μg/mL, respectively). Methanol extracts were the best for scavenging superoxide anion radicals (IC_50_ = 6.9 μg/mL) [28]. The capacity for scavenging DPPH free radicals found by other authors [68] was better in methanol leaf extracts [EC_50_ = 0.4232 mg/mL) than in ethanol (EC_50_ = 0.4872 mg/mL) or aqueous extracts (EC_50_ = 0.6554 mg/mL) in contrast to those reported by [28]. For the same concentration of samples and standard (BHT) assayed (50 μg/mL), the β-carotene bleaching activity of methanol extracts (55.20%) was also higher than the remaining samples but significantly lower than BHT (91.1%) [68]. The capacity for scavenging H_2_O_2_ was similar for both aqueous (26.02%) and methanol (28.22%) extracts but significantly lower than BHT (85.75%) when the concentration assayed in all cases was 250 μg/mL [68]. Metal chelating activity was also higher in those extracts (14.05%–14.46%) but much lower when compared to the EDTA (67.90%) [68].

The collection zone and genotype also influenced the antioxidant properties of *A. unedo* extracts of leaves [30,69]. Samples collected in Greece showed significantly higher spasmolytic activity (IC_50_ = 1.66 mg/mL) and DPPH free radical scavenging capacity (IC_50_ = 4.77 μg/mL) than the samples collected in Montenegro (IC_50_ = 5.26 mg/mL and 7.14 μg/mL, respectively). The inhibition of lipid peroxidation in liposomes induced by Fe^2+^/ascorbate and determined through thiobarbituric test activity was also higher in sample extracts from Greece, particularly when phenol content was lower [30]. The reducing power and the capacity of scavenging DPPH free radicals were evaluated in 19 genotypes of *A. unedo* collected in the Trás-os-Montes region of Portugal [69]. In reducing power and DPPH methods, the strongest (EC_50_ = 0.234 mg/mL and 0.089 mg/mL, respectively) and the weakest activities (EC_50_ = 0.378 mg/mL and 0.142 mg/mL, respectively) were found in samples collected in Vila Verde and Vila Boa 2, respectively [69].

Boulanouar *et al.* [32], studying the antioxidant activity of eight Algerian leaf extracts, measured through several *in vitro* methods, showed that *A. unedo* extract was the most active for scavenging ABTS (IC_50_ = 0.009 mg/mL), DPPH (IC_50_ = 0.006 mg/mL) and superoxide anion radicals (IC_50_ = 0.084 mg/mL).

The human erythrocyte as a cell-based model system was used to elucidate the antioxidant activity of *A. unedo* extracts [27]. In such model, the peroxidative injury in human erythrocytes is induced by AAPH [2,2'-azobis(2-amidinopropane) dihydrochloride]. The results obtained by the authors showed that leaf extracts were better to prevent peroxidation than fruit extracts. The IC_50_ values found for leaf and fruit extracts were 0.062 and 0.430 mg of extract/mL, respectively, after 3 h of incubation, but significantly lower than that obtained with ascorbic acid, used as standard (0.031 mg/mL). 

According to some authors [23], leaf extracts of *A. unedo* may be used for the treatment and/or prevention of cardiovascular diseases because their studies demonstrated that such samples reduced store-operated Ca^2+^ entry induced by thrombin or by selective depletion of the two Ca^2+^ stores in platelets, the dense tubular system and the acidic stores. The same extracts were also able to reduce both basal and thrombin-stimulated protein tyrosine phosphorylation. Therefore, the extracts have *in vitro* anti-aggregant effects in human platelets. An anti-aggregant effect was also reported by [76] but in rat platelets. The authors reported that tannins isolated from the methanol extract presented a strong anti-platelet effect and therefore responsible for such action.

Ethanol extracts of *A. unedo* leaves collected in Greece and Montenegro possessed spasmolytic activities in rat ileum, probably mediated via the inhibition of Ca channels. The authors attributed this action to the relative high contents of phenols, tannins, arbutin and flavonoids [30]. Extracts rich in oligomeric condensed tannins and catechin gallates possessed an endothelium-dependent, vasorelaxant activity. Pre-contracted aortic rings, previously removed from male Wistar rats, with noradrenaline, relaxed strongly in the presence of *A. unedo* extracts [77].

### 3.3. Roots

The aqueous extract of roots had antibacterial activity on *Escherichia coli* comparable to that of piperacilline (diamter of inhibition zone = 30 mm in both cases) [54]. Moderate antibacterial activity was reported by [78] for water extract and phenolic fractions of *A. unedo* collected in Tlemcen (Algeria) against *E. coli* (MIC = 200 μg/mL) and *Staphylococcus aureus* (MIC = 200 μg/mL for a fraction obtained by column chromatography after elution with 50% methanol/water (v/v) containing 0.1% (v/v) acetic acid. Such activity could be attributed to the secondary metabolites detected in samples such as quinones, anthraquinones, flavonoids, tannins and anthocyanins [78].

The biological properties of root extracts from diverse locations are largely referred. Hypoglycemiant effect of aqueous extracts of *A. unedo* from Morocco in neonatal streptozotocin-induced diabetic rats under chronic treatment [79]. Chronic treatment with aqueous extracts of *A. unedo* roots reduced hypertension development; prevented the myocardial hypertrophy; ameliorated vascular reactivity and renal functional parameters caused by l-NG-Nitroarginine methyl ester (L-NAME) of male Wistar rats [80]. The extracts also improved the sensitivity of the arterial baroreceptor controlling the heart rate to acute increases of arterial pressure [80].

### 3.4. Entire Plant

Anti-inflammatory activity of entire plant extracts of strawberry tree was also reported by Carcache-Blanco *et al.* [57], through the inhibition of cyclooxygenase-2 (COX-2). This property was more evident for some compounds isolated from extracts: 7β-hydroxystigmast-4-en-3-one, (−)-catechin, lupeol, betulinic acid, and α-amyrin acetate.

## 4. Other Products Obtained from *A. unedo*

### 4.1. Honey

**Figure 8:**
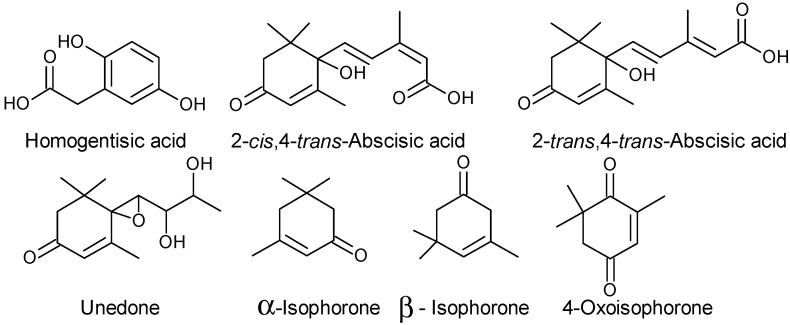
Chemical structures of markers of the botanical origin of strawberry tree honey.

Strawberry tree honey is a known product of Mediterranean regions which is characterized for its “bitter taste” [81]. Homogentisic acid (2,5-dihydroxyphenylacetic) has been considered as a chemical marker of the botanical origin of strawberry tree honey [82,83] along with (±)-2-*cis*,4-*trans*-abscisic acid (*c,t*-ABA), (±)-2-t*rans*, 4-*trans*-abscisic acid (*t,t*-ABA) and unedone [84]. From the aromatic profile of strawberry tree honey from Sardinia (Italy) and Canary islands (Spain) some authors [85,86] reported that norisoprenoids such as α-isophorone, β-isophorone and 4-oxoisophorone (Figure 8) could be considered specific floral origin markers. Proline was found to be the main free amino acid, followed by glutamic acid, arginine, alanine and phenylalanine in strawberry tree honey [87]. The antioxidant and anti-radical activities [88,89,90,91] of strawberry tree honey have been attributed to the homogentisic acid [91].

### 4.2. Spirit Beverages

The spirit beverage that comes from the distillation of fermented fruits of *A. unedo* has also been target of chemical surveys. In Portugal, this distilled beverage is known as “aguardente de medronho” [92]; in Italy as “Corbezzolo” [93], and in Greece as “Koumaro” [18]. In Portugal, two denominations of origin distillates are already recognized: *Medronho do Algarve* e *Medronho do Buçaco* [94].

In Portugal, “aguardente de medronho” has as main constituents methanol and ethyl acetate, nevertheless the levels of methanol never exceeded the concentrations prescribed by law [94]. Alcohols, esters, acids and aromatic compounds constituted the main group of compounds in “koumaro” [18]. Methanol, ethyl acetate, isovaleric acid and *trans*-anethole were the main components in every group, respectively [18]. In all samples studied by the authors, two of them had concentrations of methanol higher than those permitted by law (1000 d/hL absolute alcohol) [94]. Calcium was the main mineral in the same samples [18].

Methanol and ethyl acetate also predominated in distilled alcoholic beverages obtained from solid-state fermentation of strawberry tree fruits from Spain [95]. In the same work, the authors concluded that the addition of the ethanol-producing yeast (*Saccharomyces cerevisiae* IFI83) led to a more efficient utilization of the reducing sugars for ethanol production than did the indigenous microbiota of the fruits in the spontaneous fermentations [96].

Sardinian strawberry tree distillate is characterised by a lower content of C_7_ to C_9_ primary alcohols and in general by a higher content of C_6_ to C_10_ ethyl esters and C_12_ to C_18_ fatty esters when compared to those from Greece and Portugal [18,94,95].

The quality of distilled product may be affected by off-flavours when uncontrolled fermentations occur, therefore the nature of yeasts present in musts contribute to the sensory characteristics [93]. These authors studied the diversity of the yeast population and the killer activity of *S. cerevisiae* isolates obtained during the fermentation period of arbutus fruits from Portugal. They found a diversity of autochthonous yeasts that fermented arbutus fruits at room temperature through a solid state process. As fermentation progressed, a microbial succession is observed, with the final prevalence of *S. cerevisiae* [93].

New aromatic pomegranate liquor obtained by maceration of pomegranate juice and arils from Portuguese origins (‘Assaria’) in *A. unedo* distillate was performed by [97]. The authors found that anthocyanins of pomegranate juice undergo degradation during the maceration, particularly the monoglucosides, while ellagitannin compounds remained stable [97]. Most strawberry tree fruit spirit volatiles (ethanal, ethyl acetate and 1-hexanol) were also detected in the liquor but in lower amounts than in the spirit and the volatiles of pomegranate origin (limonene, 1-hexenol and *trans*-caryophyllene) also had little contribution to the volatile profile of the liquor [97].

## 5. Conclusions

From ancient times, leaves and fruits of *A. unedo* L. have been used in folk medicine. Fruits have also been used for making brandies, jams, jellies and marmalades, and less frequently eaten as fresh fruit. The chemical composition of fruits and leaves, in which phenolic acids, flavonoids, tannins, anthocyanins, vitamins are present, may be responsible for the reported biological properties. More recently, the antioxidant activity of fruit extracts has also been used in meat industry to preserve the quality and prevent oxidation of lipids and proteins. *Arbutus* honey has also been chemically characterized and specific floral origin markers have been tentatively found. The biological properties of honey, particularly the antioxidant ability, have been attributed to their phenols and even to homogentisic acid, one of the possible chemical markers. The chemical characterization of distilled beverage obtained from fruits of *A. unedo* has been established and regulated. Studies regarding the increased shelf life periods of fruits started recently. All of these approaches have as main goal to increase the utilization of fruits and/or derivatives in human nutrition due to their beneficial properties to human health.
